# 2-Amino-4-(2-chloro­phen­yl)-5-oxo-5,6,7,8-tetra­hydro-4*H*-chromene-3-carbonitrile ethanol monosolvate

**DOI:** 10.1107/S1600536811043650

**Published:** 2011-10-29

**Authors:** Yan Qiao, Lingqian Kong, Guifang Chen, Shengli Li, Zhiqing Gao

**Affiliations:** a Dongchang College, Liaocheng University, Liaocheng 250059, People’s Republic of China; b College of Chemistry and Chemical Engineering, Liaocheng University, Shandong 252059, People’s Republic of China

## Abstract

In the title compound, C_16_H_13_ClN_2_O_2_·C_2_H_6_O, the fused cyclo­hexene and pyran rings adopt envelope and flattened boat conformations, respectively. In the crystal, N—H⋯O and O—H⋯O hydrogen bonds link the chromene and ethanol solvent mol­ecules into infinite chains along the *c* axis, and N—H⋯N hydrogen bonds link these chains into a three-dimensional framework. Weak C—H⋯π inter­actions are also present.

## Related literature

For the background, see: Lokaj *et al.* (1990 ▶); Marco *et al.* (1993 ▶). For crystal structures similar to the title compound, see: Tu *et al.* (2001 ▶).
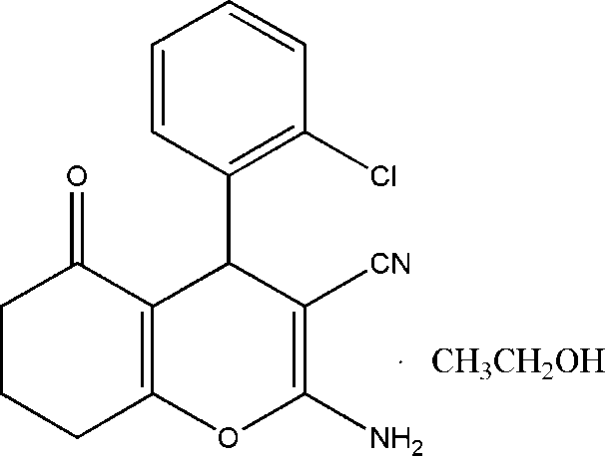


## Experimental

### 

#### Crystal data

C_16_H_13_ClN_2_O_2_·C_2_H_6_O
*M* *_r_* = 346.80
Triclinic, 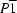

*a* = 8.7610 (8) Å
*b* = 9.6281 (9) Å
*c* = 10.7951 (11) Å
α = 76.878 (1)°
β = 83.028 (2)°
γ = 77.632 (1)°
*V* = 863.69 (14) Å^3^
*Z* = 2
Mo *K*α radiation
μ = 0.24 mm^−1^
*T* = 298 K
0.47 × 0.46 × 0.21 mm

#### Data collection

Bruker SMART APEX CCD area-detector diffractometer
Absorption correction: multi-scan (*SADABS*; Sheldrick, 1996 ▶) *T*_min_ = 0.896, *T*_max_ = 0.952
4606 measured reflections
3003 independent reflections
1428 reflections with *I* > 2σ(*I*)
*R*_int_ = 0.057

#### Refinement

*R*[*F* ^2^ > 2σ(*F* ^2^)] = 0.082
*wR*(*F* ^2^) = 0.230
*S* = 0.90
3003 reflections
219 parameters
1 restraint
H-atom parameters constrained
Δρ_max_ = 0.36 e Å^−3^
Δρ_min_ = −0.31 e Å^−3^

### 

Data collection: *SMART* (Siemens, 1996 ▶); cell refinement: *SAINT* (Siemens, 1996 ▶); data reduction: *SAINT*; program(s) used to solve structure: *SHELXS97* (Sheldrick, 2008 ▶); program(s) used to refine structure: *SHELXL97* (Sheldrick, 2008 ▶); molecular graphics: *SHELXTL* (Sheldrick, 2008 ▶); software used to prepare material for publication: *SHELXTL*.

## Supplementary Material

Crystal structure: contains datablock(s) I, global. DOI: 10.1107/S1600536811043650/bq2312sup1.cif

Structure factors: contains datablock(s) I. DOI: 10.1107/S1600536811043650/bq2312Isup2.hkl

Supplementary material file. DOI: 10.1107/S1600536811043650/bq2312Isup3.cml

Additional supplementary materials:  crystallographic information; 3D view; checkCIF report

## Figures and Tables

**Table 1:** Hydrogen-bond geometry (Å, °) *Cg* is the centroid of the C1–C6 ring.

*D*—H⋯*A*	*D*—H	H⋯*A*	*D*⋯*A*	*D*—H⋯*A*
N2—H2*A*⋯N1^i^	0.86	2.19	3.037 (5)	167
N2—H2*B*⋯O3	0.86	1.99	2.851 (5)	178
O3—H3⋯O1^ii^	0.82	1.97	2.765 (5)	164
C14—H14*B*⋯*Cg*^iii^	0.97	2.96	3.704 (5)	135

Symmetry codes: (i) 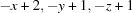; (ii) 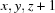; (iii) 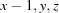.

**Fig. 1.**
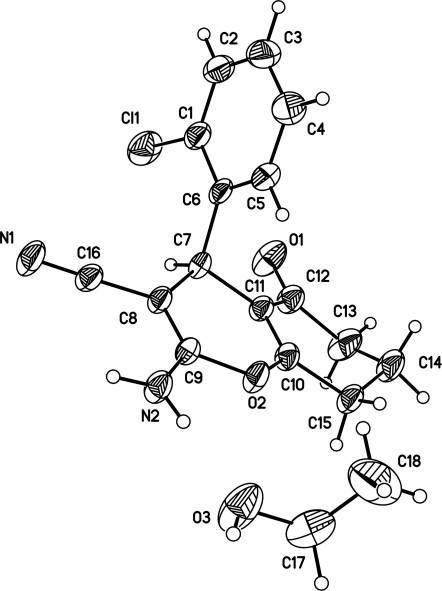
The title molecule with the atomic numbering scheme. The displacement ellipsoids are shown at the 30% probability level.

**Fig. 2.**
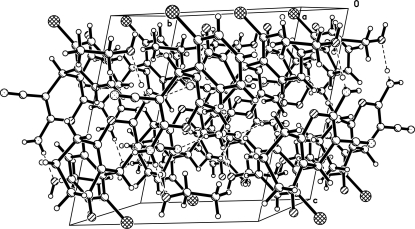
The packing of the title compound. N-H···N, N-H···O and O-H···O interactions are represented with dashed lines.

## supplementary crystallographic information

### Comment

The present investigation is a continuation of our work that includes syntheses and structural studies of polyfunctionalized substituted pyran derivatives, owing to their  biological activities (Lokaj *et al.*, 1990; Marco *et al.*, 1993).We obtained the title compound, (I), and reported here its crystal structure in the paper.

In the crystal structure, it is observed that structure unit contains a substituted 5,6,7,8-tetrahydro-4H-chromene, a benzene ring and a ethanol solvate. The pyran ring adopts a sofa conformation, the dihedral angle between the (O2/C8-C11) plane and the C8/C7/C11 plane is 16.14 (4)°. Meanwhile, the (O2/C8-C11) plane forms an angle of  88.55 (13)° with the phenyl plane (C1-C6), which means that the pyran ring and the benzene ring is nearly perpendicular. In the crystal, the nitrile group is typical [N≡C = 1.148 (5)Å] and the carbonyl group also is reasonable [C═O =1.223 (6)Å].

Moreover, the plane (C10-C15) also adopts an chair configuration in the compound, and the dihedral angle between the (C10-C15) plane and the (C13-C15) plane is 46.19 (5)°.

In (I) (Fig. 1), the bond lengths and angles of the main molecule are normal and correspond to those observed in 2-amino-7,7-dimethyl-5-oxo-4-phenyl- 5,6,7,8-tetra-hydro-4H-chromene-3-carbonitrile (Tu *et al.*, 2001).

In the crystal structure, there exist typical intermolecular N-H···N, N—H···O, O—H···O hydrogen bonds and weak C-H···π  interactions (Table 1.). Intermolecular N-H···O and O—H···O hydrogen bonds link the molecules and ethanol solvent into infinite chain along c-axis and intermolecular hydrogen bonds link these chains forming three-dimensional framework.

### Experimental

Malononitrile (5 mmol), 1,3-cyclohexanedione (5 mmol) and 2-chorobenzaldehyde (5 mmol) was dissolved in 20 ml DMF in a round-bottom flask. The mixture was warmed, with agitation, to 423 K over a period of  6 h. The resulting solution was cooled. Crystal of (I) suitable for X-ray diffraction analysis were obtained by recrystallized from ethanol.

### Refinement

All H atoms were placed in geometrically idealized positions (N-H 0.86, O-H 0.82 and C-H 0.93-0.98 Å ) and treated as riding on their parent atoms, with U_iso_(H) = 1.2-1.5U_eq_(C) (C, O, N).

### Crystal data

**Table:**

C_16_H_13_ClN_2_O_2_·C_2_H_6_O	*Z* = 2
*M**_r_* = 346.80	*F*(000) = 364
Triclinic, *P*1	*D*_x_ = 1.334 Mg m^−^^3^
Hall symbol: -P 1	Mo *K*α radiation, λ = 0.71073 Å
*a* = 8.7610 (8) Å	Cell parameters from 821 reflections
*b* = 9.6281 (9) Å	θ = 2.6–25.4°
*c* = 10.7951 (11) Å	µ = 0.24 mm^−^^1^
α = 76.878 (1)°	*T* = 298 K
β = 83.028 (2)°	Block, red
γ = 77.632 (1)°	0.47 × 0.46 × 0.21 mm
*V* = 863.69 (14) Å^3^	

### Data collection

**Table:**

Bruker SMART APEX CCD area-detector diffractometer	3003 independent reflections
Radiation source: fine-focus sealed tube	1428 reflections with *I* > 2σ(*I*)
graphite	*R*_int_ = 0.057
phi and ω scans	θ_max_ = 25.0°, θ_min_ = 1.9°
Absorption correction: multi-scan (*SADABS*; Sheldrick, 1996)	*h* = −10→10
*T*_min_ = 0.896, *T*_max_ = 0.952	*k* = −9→11
4606 measured reflections	*l* = −12→12

### Refinement

**Table:**

Refinement on *F*^2^	Primary atom site location: structure-invariant direct methods
Least-squares matrix: full	Secondary atom site location: difference Fourier map
*R*[*F*^2^ > 2σ(*F*^2^)] = 0.082	Hydrogen site location: inferred from neighbouring sites
*wR*(*F*^2^) = 0.230	H-atom parameters constrained
*S* = 0.90	*w* = 1/[σ^2^(*F*_o_^2^) + (0.126*P*)^2^] where *P* = (*F*_o_^2^ + 2*F*_c_^2^)/3
3003 reflections	(Δ/σ)_max_ = 0.001
219 parameters	Δρ_max_ = 0.36 e Å^−^^3^
1 restraint	Δρ_min_ = −0.31 e Å^−^^3^

### Special details

**Table:**

Geometry. All esds (except the esd in the dihedral angle between two l.s. planes) are estimated using the full covariance matrix. The cell esds are taken into account individually in the estimation of esds in distances, angles and torsion angles; correlations between esds in cell parameters are only used when they are defined by crystal symmetry. An approximate (isotropic) treatment of cell esds is used for estimating esds involving l.s. planes.
Refinement. Refinement of F^2^ against ALL reflections. The weighted R-factor wR and goodness of fit S are based on F^2^, conventional R-factors R are based on F, with F set to zero for negative F^2^. The threshold expression of F^2^ > 2sigma(F^2^) is used only for calculating R-factors(gt) etc. and is not relevant to the choice of reflections for refinement. R-factors based on F^2^ are statistically about twice as large as those based on F, and R- factors based on ALL data will be even larger.

### Fractional atomic coordinates and isotropic or equivalent isotropic displacement parameters (Å^2^)

**Table:**

	*x*	*y*	*z*	*U*_iso_*/*U*_eq_	
Cl1	0.94359 (18)	0.32844 (18)	0.01378 (14)	0.0853 (6)	
N1	1.0042 (5)	0.4987 (5)	0.3508 (4)	0.0694 (13)	
N2	0.7142 (4)	0.3876 (4)	0.6036 (4)	0.0543 (11)	
H2A	0.7912	0.4295	0.6051	0.065*	
H2B	0.6527	0.3694	0.6711	0.065*	
O1	0.5581 (4)	0.2859 (5)	0.0845 (3)	0.0796 (12)	
O2	0.5602 (3)	0.2879 (3)	0.5179 (3)	0.0531 (9)	
O3	0.5099 (5)	0.3347 (6)	0.8285 (4)	0.1065 (16)	
H3	0.5180	0.3377	0.9026	0.160*	
C1	0.9531 (5)	0.1785 (6)	0.1361 (5)	0.0551 (13)	
C2	1.0586 (6)	0.0538 (7)	0.1199 (6)	0.0659 (15)	
H2	1.1212	0.0522	0.0441	0.079*	
C3	1.0716 (6)	−0.0672 (7)	0.2153 (6)	0.0736 (16)	
H3A	1.1434	−0.1508	0.2047	0.088*	
C4	0.9784 (6)	−0.0667 (6)	0.3281 (6)	0.0707 (16)	
H4	0.9861	−0.1494	0.3930	0.085*	
C5	0.8745 (5)	0.0572 (5)	0.3432 (5)	0.0532 (12)	
H5	0.8139	0.0577	0.4201	0.064*	
C6	0.8560 (4)	0.1819 (5)	0.2487 (4)	0.0443 (11)	
C7	0.7362 (4)	0.3145 (5)	0.2726 (4)	0.0429 (11)	
H7	0.7373	0.3918	0.1960	0.051*	
C8	0.7721 (5)	0.3698 (5)	0.3836 (4)	0.0437 (11)	
C9	0.6906 (5)	0.3513 (5)	0.4982 (4)	0.0437 (11)	
C10	0.5007 (5)	0.2644 (5)	0.4148 (4)	0.0454 (11)	
C11	0.5740 (5)	0.2806 (5)	0.2992 (4)	0.0433 (11)	
C12	0.4933 (5)	0.2721 (6)	0.1923 (5)	0.0561 (13)	
C13	0.3260 (5)	0.2516 (7)	0.2181 (5)	0.0745 (17)	
H13A	0.3012	0.2056	0.1539	0.089*	
H13B	0.2570	0.3459	0.2115	0.089*	
C14	0.2971 (6)	0.1595 (6)	0.3488 (5)	0.0670 (15)	
H14A	0.3567	0.0616	0.3523	0.080*	
H14B	0.1868	0.1544	0.3640	0.080*	
C15	0.3445 (5)	0.2227 (5)	0.4516 (5)	0.0541 (13)	
H15A	0.2668	0.3076	0.4644	0.065*	
H15B	0.3487	0.1515	0.5313	0.065*	
C16	0.8995 (5)	0.4412 (5)	0.3669 (4)	0.0489 (12)	
C17	0.3742 (7)	0.2814 (7)	0.8216 (6)	0.097 (2)	
H17A	0.3331	0.3234	0.7390	0.117*	
H17B	0.2949	0.3108	0.8865	0.117*	
C18	0.4070 (11)	0.1237 (8)	0.8401 (7)	0.136 (3)	
H18A	0.4878	0.0942	0.7776	0.204*	
H18B	0.3137	0.0910	0.8308	0.204*	
H18C	0.4412	0.0817	0.9240	0.204*	

### Atomic displacement parameters (Å^2^)

**Table:**

	*U*^11^	*U*^22^	*U*^33^	*U*^12^	*U*^13^	*U*^23^
Cl1	0.0840 (11)	0.0960 (12)	0.0676 (10)	−0.0272 (9)	0.0204 (7)	−0.0043 (8)
N1	0.044 (2)	0.100 (4)	0.076 (3)	−0.036 (2)	0.001 (2)	−0.025 (3)
N2	0.046 (2)	0.078 (3)	0.050 (3)	−0.029 (2)	−0.0001 (18)	−0.021 (2)
O1	0.060 (2)	0.137 (4)	0.052 (2)	−0.040 (2)	−0.0035 (18)	−0.023 (2)
O2	0.0437 (17)	0.074 (2)	0.050 (2)	−0.0305 (16)	0.0028 (14)	−0.0154 (17)
O3	0.096 (3)	0.183 (5)	0.057 (3)	−0.070 (3)	0.008 (2)	−0.025 (3)
C1	0.041 (3)	0.071 (4)	0.062 (3)	−0.020 (3)	0.003 (2)	−0.026 (3)
C2	0.048 (3)	0.086 (4)	0.075 (4)	−0.018 (3)	0.009 (3)	−0.041 (4)
C3	0.050 (3)	0.074 (4)	0.101 (5)	−0.003 (3)	−0.005 (3)	−0.036 (4)
C4	0.058 (3)	0.069 (4)	0.084 (4)	−0.008 (3)	−0.010 (3)	−0.014 (3)
C5	0.037 (3)	0.061 (3)	0.061 (3)	−0.012 (2)	0.003 (2)	−0.014 (3)
C6	0.027 (2)	0.059 (3)	0.054 (3)	−0.018 (2)	0.0017 (19)	−0.018 (2)
C7	0.031 (2)	0.051 (3)	0.049 (3)	−0.016 (2)	0.0002 (18)	−0.008 (2)
C8	0.033 (2)	0.056 (3)	0.047 (3)	−0.017 (2)	−0.0002 (19)	−0.013 (2)
C9	0.031 (2)	0.050 (3)	0.053 (3)	−0.013 (2)	−0.0028 (19)	−0.012 (2)
C10	0.037 (2)	0.054 (3)	0.051 (3)	−0.017 (2)	−0.006 (2)	−0.015 (2)
C11	0.036 (2)	0.054 (3)	0.045 (3)	−0.015 (2)	0.000 (2)	−0.017 (2)
C12	0.045 (3)	0.074 (4)	0.057 (3)	−0.019 (2)	−0.004 (2)	−0.020 (3)
C13	0.038 (3)	0.125 (5)	0.074 (4)	−0.027 (3)	−0.010 (2)	−0.037 (4)
C14	0.043 (3)	0.091 (4)	0.079 (4)	−0.032 (3)	−0.001 (2)	−0.026 (3)
C15	0.036 (3)	0.068 (3)	0.062 (3)	−0.020 (2)	0.002 (2)	−0.015 (3)
C16	0.035 (2)	0.064 (3)	0.052 (3)	−0.014 (2)	−0.001 (2)	−0.018 (2)
C17	0.068 (4)	0.131 (7)	0.085 (5)	−0.004 (4)	0.002 (3)	−0.021 (4)
C18	0.203 (10)	0.088 (6)	0.102 (6)	−0.006 (6)	0.011 (6)	−0.020 (5)

### Geometric parameters (Å, °)

**Table:**

Cl1—C1	1.716 (5)	C6—CG	1.405 (4)
N1—C16	1.146 (5)	C6—C7	1.519 (6)
N2—C9	1.315 (5)	C7—C11	1.505 (5)
N2—H2A	0.8600	C7—C8	1.507 (6)
N2—H2B	0.8600	C7—H7	0.9800
O1—C12	1.223 (5)	C8—C9	1.346 (6)
O2—C10	1.367 (5)	C8—C16	1.405 (6)
O2—C9	1.379 (5)	C10—C11	1.324 (6)
O3—C17	1.407 (7)	C10—C15	1.491 (6)
O3—H3	0.8200	C11—C12	1.450 (6)
C1—CG	1.373 (5)	C12—C13	1.505 (6)
C1—C2	1.381 (7)	C13—C14	1.511 (7)
C1—C6	1.399 (6)	C13—H13A	0.9700
C2—C3	1.363 (7)	C13—H13B	0.9700
C2—CG	1.376 (5)	C14—C15	1.518 (6)
C2—H2	0.9300	C14—H14A	0.9700
C3—CG	1.376 (6)	C14—H14B	0.9700
C3—C4	1.382 (8)	C15—H15A	0.9700
C3—H3A	0.9300	C15—H15B	0.9700
C4—C5	1.366 (7)	C17—C18	1.455 (7)
C4—CG	1.380 (6)	C17—H17A	0.9700
C4—H4	0.9300	C17—H17B	0.9700
C5—CG	1.359 (5)	C18—H18A	0.9600
C5—C6	1.381 (6)	C18—H18B	0.9600
C5—H5	0.9300	C18—H18C	0.9600
			
C9—N2—H2A	120.0	C11—C10—C15	126.5 (4)
C9—N2—H2B	120.0	O2—C10—C15	110.5 (4)
H2A—N2—H2B	120.0	C10—C11—C12	119.2 (4)
C10—O2—C9	118.7 (3)	C10—C11—C7	122.6 (4)
C17—O3—H3	109.5	C12—C11—C7	118.1 (4)
CG—C1—C2	60.0 (3)	O1—C12—C11	120.8 (4)
CG—C1—C6	60.9 (3)	O1—C12—C13	121.4 (4)
C2—C1—C6	120.8 (5)	C11—C12—C13	117.7 (4)
CG—C1—Cl1	178.0 (4)	C12—C13—C14	112.0 (4)
C2—C1—Cl1	118.1 (4)	C12—C13—H13A	109.2
C6—C1—Cl1	121.1 (4)	C14—C13—H13A	109.2
C3—C2—CG	60.3 (3)	C12—C13—H13B	109.2
C3—C2—C1	120.0 (5)	C14—C13—H13B	109.2
CG—C2—C1	59.7 (3)	H13A—C13—H13B	107.9
C3—C2—H2	119.8	C13—C14—C15	110.9 (4)
CG—C2—H2	179.7	C13—C14—H14A	109.5
C1—C2—H2	120.1	C15—C14—H14A	109.5
C2—C3—CG	60.3 (3)	C13—C14—H14B	109.5
C2—C3—C4	120.4 (5)	C15—C14—H14B	109.5
CG—C3—C4	60.1 (3)	H14A—C14—H14B	108.1
C2—C3—H3A	120.0	C10—C15—C14	110.6 (4)
CG—C3—H3A	179.6	C10—C15—H15A	109.5
C4—C3—H3A	119.6	C14—C15—H15A	109.5
C5—C4—CG	59.3 (3)	C10—C15—H15B	109.5
C5—C4—C3	119.1 (6)	C14—C15—H15B	109.5
CG—C4—C3	59.7 (4)	H15A—C15—H15B	108.1
C5—C4—H4	120.3	N1—C16—C8	178.5 (5)
CG—C4—H4	179.5	O3—C17—C18	111.4 (6)
C3—C4—H4	120.6	O3—C17—H17A	109.3
CG—C5—C4	60.9 (3)	C18—C17—H17A	109.3
CG—C5—C6	61.7 (3)	O3—C17—H17B	109.3
C4—C5—C6	122.6 (5)	C18—C17—H17B	109.3
CG—C5—H5	179.0	H17A—C17—H17B	108.0
C4—C5—H5	118.7	C17—C18—H18A	109.5
C6—C5—H5	118.8	C17—C18—H18B	109.5
C5—C6—C1	117.0 (4)	H18A—C18—H18B	109.5
C5—C6—CG	58.4 (3)	C17—C18—H18C	109.5
C1—C6—CG	58.6 (3)	H18A—C18—H18C	109.5
C5—C6—C7	119.2 (4)	H18B—C18—H18C	109.5
C1—C6—C7	123.7 (4)	C5—CG—C1	120.4 (3)
CG—C6—C7	177.6 (4)	C5—CG—C3	120.0 (4)
C11—C7—C8	108.2 (3)	C1—CG—C3	119.7 (3)
C11—C7—C6	110.9 (3)	C5—CG—C2	179.3 (3)
C8—C7—C6	112.1 (3)	C1—CG—C2	60.3 (3)
C11—C7—H7	108.5	C3—CG—C2	59.4 (3)
C8—C7—H7	108.5	C5—CG—C4	59.8 (3)
C6—C7—H7	108.5	C1—CG—C4	179.8 (3)
C9—C8—C16	118.6 (4)	C3—CG—C4	60.2 (3)
C9—C8—C7	123.3 (4)	C2—CG—C4	119.5 (3)
C16—C8—C7	118.0 (4)	C5—CG—C6	59.9 (3)
N2—C9—C8	129.3 (4)	C1—CG—C6	60.5 (3)
N2—C9—O2	109.7 (4)	C3—CG—C6	179.6 (3)
C8—C9—O2	121.0 (4)	C2—CG—C6	120.8 (3)
C11—C10—O2	123.0 (4)	C4—CG—C6	119.7 (3)

### Hydrogen-bond geometry (Å, °)

**Table:**

*D*—H···*A*	*D*—H	H···*A*	*D*···*A*	*D*—H···*A*
N2—H2A···N1^i^	0.86	2.19	3.037 (5)	167.
N2—H2B···O3	0.86	1.99	2.851 (5)	178.
O3—H3···O1^ii^	0.82	1.97	2.765 (5)	164.
C14—H14B···CG^iii^	0.97	2.96	3.704 (5)	135.

Symmetry codes: (i) −*x*+2, −*y*+1, −*z*+1; (ii) *x*, *y*, *z*+1; (iii) *x*−1, *y*, *z*.
